# UNICORN: a deep learning model for integrating multi-stain data in histopathology

**DOI:** 10.1038/s41746-026-02829-6

**Published:** 2026-06-18

**Authors:** Valentin Koch, Sabine Bauer, Shweta Mahajan, Valerio Lupperger, Michael Joner, Heribert Schunkert, Julia A. Schnabel, Moritz von Scheidt, Carsten Marr

**Affiliations:** 1 Computational Health Center, Helmholtz Munich - German Research Center for Environmental Health, Munich, Germany; 2 School of Computation and Information Technology, Technical University of Munich, Munich, Germany; 3 Department of Cardiology, German Heart Centre, TUM University Hospital, Munich, Germany; 4 DZHK (German Center for Cardiovascular Research), Partner Site Munich Heart Alliance, Munich, Germany; 5 MLL Munich Leukemia Laboratory, Munich, Germany; 6 School of Biomedical Engineering and Imaging Sciences, King’s College London, London, UK; 7 Department of Medicine III, Ludwig Maximilian University Hospital, Munich, Germany; 8 Department of Physics, Ludwig-Maximilians-Universität München, Munich, Germany; 9 DKTK, German Cancer Consortium, Heidelberg, Germany

**Keywords:** Computational biology and bioinformatics, Diseases

## Abstract

The integration of multi-stain histopathology images through deep learning poses a significant challenge. Current approaches struggle with data heterogeneity and missing data, as concatenating multi-stain features may not effectively model stain-specific and cross-stain interactions. We introduce UNICORN (UNiversal stain Integration network for CORonary classificatioN), a two-stage, end-to-end trainable model comprising transformer self-attention blocks to process multi-stain histopathology for atherosclerosis severity prediction. The initial stage employs domain-specific expert models to extract features from each staining. An aggregation expert model then integrates features by learning their interactions. On a multi-class, multi-stain whole slide images (WSIs) dataset of atherosclerotic lesions from Munich Cardiovascular Studies Biobank (MISSION), UNICORN achieved a classification accuracy of 0.68, significantly outperforming state-of-the-art models. UNICORN identifies relevant tissue phenotypes across stainings and implicitly models disease progression. Its explainability and effectiveness in predicting atherosclerosis progression highlight the potential for broader applications in medical research and decision support.

## Introduction

Atherosclerosis, a complex inflammatory disease of the arterial wall, is a leading cause of cardiovascular morbidity and mortality worldwide. Histopathological classification of atherosclerosis, as proposed by Stary et al.^1,2^ and adapted by Virmani et al.^3,4^, plays a crucial role in assessing disease severity, progression and potential therapeutic interventions. This classification scheme assesses features such as the thickness of the fibrous cap, the presence of a necrotic core, the degree of inflammation and the extent of calcification within arterial plaques^5–7^.

Whole slide imaging (WSI) allows detailed visualization of tissue samples at micrometer resolution, providing critical insight into various medical conditions. While the most commonly used staining method, haematoxylin and eosin (H&E), provides a tissue overview and is used to diagnose many diseases, in some cases, further stainings are required to fully understand underlying tissue characteristics^8^. For example, immunohistochemistry (IHC) staining is essential for cancer detection, providing critical prognostic, diagnostic, and therapeutic information that enables precise and personalized treatment strategies^9^. Specific staining methods, like von Kossa silver stain or Movat pentachrome stain, highlight particular tissue components, such as mineralization or connective tissue composition and lipid distribution^10^. Manual integration and interpretation of multi-stain data is challenging, as multiple high-resolution images that are each potentially gigabytes in size need to be examined in detail.

In recent years, computational pathology focusing on the analysis of digitized pathology slides, has seen remarkable advances largely driven by modern machine learning techniques, especially deep learning. These advances cover several areas, including disease classification, tissue segmentation, and mutation prediction^11–14^. Given the large size of WSIs, a common strategy is to decompose them into manageable-sized image patches. Subsequently, a pre-trained feature extractor condenses each patch into a low-dimensional feature vector, which is then processed by a Multiple Instance Learning (MIL) network^15,16^. Recent developments in transformer architectures, in particular Vision Transformers (ViT), have shown promising results as the backbone of state-of-the-art feature extractors^17–19^, and when applied as WSI classification networks^11^.

Although there has been a lot of research on cancer prediction and using deep learning on WSIs, computational pathology is quite new in the field of atherosclerosis. Holmberg et al. used co-registered histopathology and Optical Coherence Tomography (OCT) images to improve segmentation of calcification and lesions in OCT images using a UNet^20^. The open-source tool Vesseg has been developed to segment lesions using U-nets on H&E-stained brachiocephalic arteries^21^.

We introduce UNICORN, a two-stage, end-to-end trainable transformer architecture capable of handling different stains as input, in particular, features obtained from WSIs with various staining protocols. The first stage captures stain-specific features, while the second stage models inter-stain interactions, providing an advantage over simply concatenating features from multiple stains^22,23^. To our knowledge, this is the first AI model for multi-stain histopathological classification for atherosclerosis. We show how UNICORN implicitly captures disease progression in atherosclerosis, handles missing data effectively during both training and inference, and can be robustly applied in real-world settings.

## Results

### UNICORN architecture integrates different tissue stainings

**Fig. 1:**
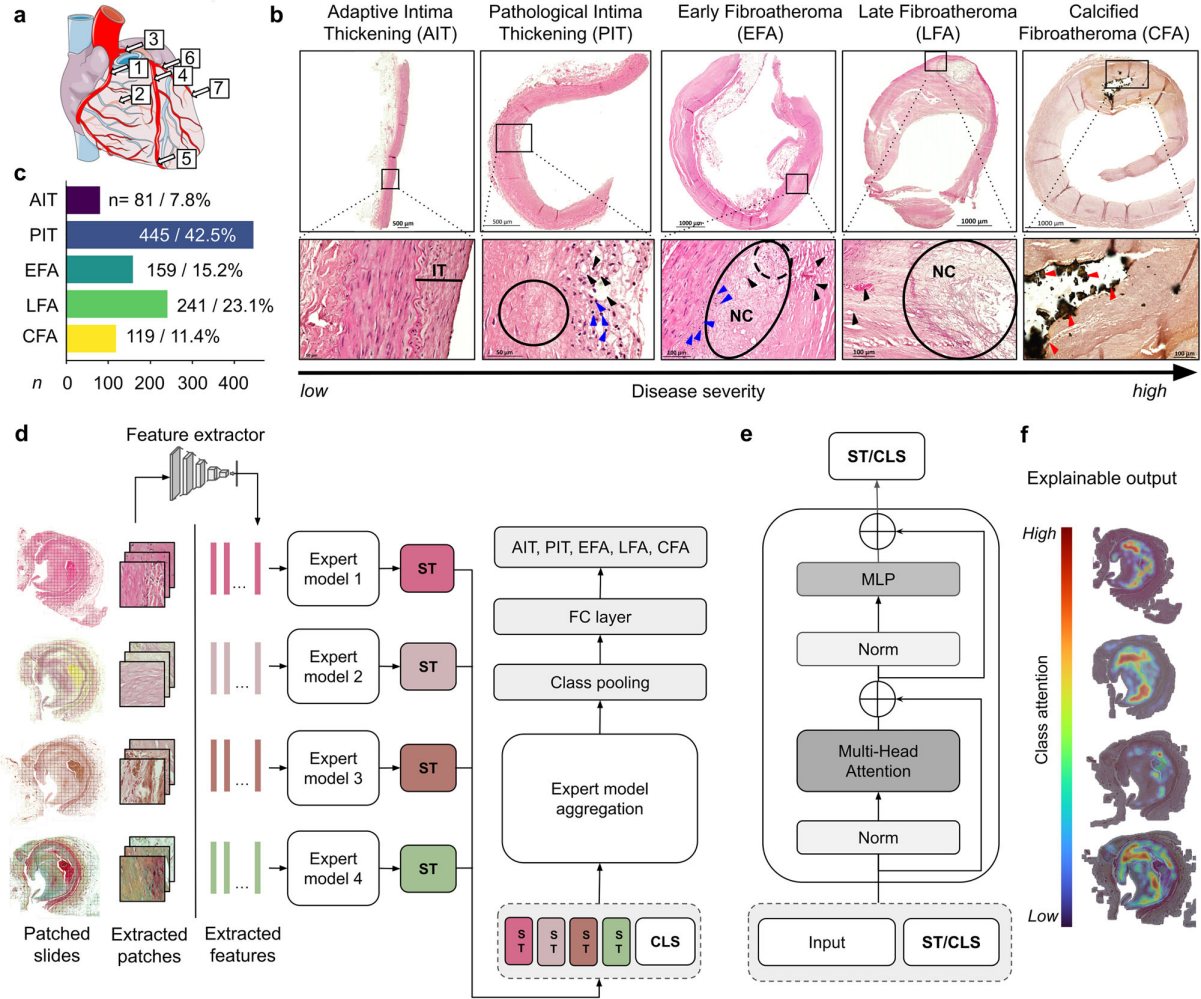
UNICORN architecture and the MISSION biobank. **a** MISSION coronary artery segments used in this study. 1 + 2: proximal and distal parts of the right coronary artery, 3: main stem, 4 + 5: proximal and distal parts of the left coronary artery, 6 + 7: proximal and distal parts of the left circumflex coronary artery. **b** Histological classification of coronary arteries according to an adapted and simplified AHA classification based on Virmani et al.^3^. Hematoxylin and Eosin (H&E) stains are shown for AIT, PIT, EFA, and LFA, and von Kossa silver stain is shown for CFA, all exemplary with zoomed-in regions of interest. Blue and red arrows show class-specific characteristics (see main text for detailed explanations). IT: Intima thickening, NC: necrotic core. **c** Class distribution in the study cohort of MISSION consisting of *n* = 7 segments from 177 individuals. **d** UNICORN architecture: features extracted from WSIs with the four different stainings H&E, Elastica van Gieson (EvG), von Kossa (vK) and Movat pentachrome (Movat) are forwarded to four expert models consisting of two transformer blocks (e) that are specialized in processing data from a certain staining. Information from each domain is aggregated in a stain token (ST), which is used as input to the expert model aggregation, which combines information across stainings into the CLS token. The final classification score is derived from a fully connected (FC) layer that uses the CLS token as input. **e** Transformer blocks are used in expert models and for expert model aggregation (for hyperparameters see Table 2). Stain expert models take extracted features from respective stains and propagate a stain token (ST), whereas the expert model aggregation accepts the stain tokens and propagates a CLS token. MLP: multi-layer perceptron. **f** Using attention values, UNICORN can provide explainable output, highlighting regions of importance across stainings. .

UNICORN (UNiversal stain Integration network for CORonary classificatioN) is capable of integrating and processing heterogeneous data across different tissue stainings (Fig. 1). Its specialized models, each having the same architecture, include a two-layer self-attention transformer block with four attention heads (Fig. 1d). The input whole slide image is tiled into 256 × 256 px-sized patches. A pre-trained feature extractor is then used to generate embeddings for each patch (see “Methods”). Each staining is individually processed by an expert model that learns domain-specific features via patch-wise self-attention. Expert models propagate a stain token (ST) to the expert model aggregation. This component learns to aggregate the information from the four expert models in a CLS token, which is ultimately used as input to the fully connected (FC) layer that outputs a classification score. Self-attention in the expert model aggregation operates across the stain tokens. Each expert model consists of two layers of a transformer block (Fig. 1e). Architecture hyperparameters for the transformer block can be found in Table 1. Since the expert model aggregation is also a transformer that can handle a variable number of input tokens, it can process data with missing tokens during both training and inference. UNICORN is evaluated on a multi-stain, multi-class atherosclerosis dataset where it classifies five stages of coronary atherosclerosis.

**Table 1:** Hyperparameters of the transformer block in the expert models

Input dimension	512
Attention heads	4
Dimension per attention head	64
MLP dimension	512
Dropout	0.2
Positional embedding	NA

### Multi-stain atherosclerosis classification dataset

Our carefully curated Munich Cardiovascular Studies Biobank (MISSION) dataset consists of tissue sets from 177 deceased individuals with a mean age of 64 ± 17 years, 117 (66%) males and 60 (34%) females, and an average BMI of 28 ± 8. Each set contains 7 segments from different parts of the coronary tree (Fig. 1a): proximal and distal parts of the right coronary artery, main stem, proximal and distal parts of the left coronary artery, proximal and distal parts of the left circumflex coronary artery. Each segment is stained using four methods: Hematoxylin and Eosin (H&E), Elastica van Gieson (EvG), von Kossa (vK), and Movat pentachrome (Movat) stain. For lesion classification, a modified scheme according to the American Heart Association (AHA) and the classification method described by Virmani et al.^3,4^ was employed: adaptive intima thickening (AIT), pathological intima thickening (PIT), early fibroatheroma (EFA), late fibroatheroma (LFA) and calcified fibroatheroma (CFA). These labels (see “Methods” and Fig. 1) have been assigned by two biomedical experts (SB and MJ) after reviewing all four stainings. Disease severity increases from AIT over PIT, EFA, and LFA to CFA. Class-specific characteristics are highlighted in Fig. 1b: The AIT WSI shows intima thickening (IT) but intact cell structure and smooth muscle cell layer in a proteoglycan and collagen-rich matrix. PIT is described as preatherosclerotic lesions with focal fat-laden macrophages (black triangles), inflammatory cells (blue triangles) and fatty streaks. The black circle shows matrix remodeling resulting in a varying degree of smooth muscle cells. In the EFA WSI, the black circle highlights the necrotic core (NC), the blue arrows show inflammatory cells, and the black arrows point to the lipid pool. The black dotted circle shows cell debris. In the LFA case, the black circle shows the NC with cholesterol crafts and fibrocalcific surrounding, and the black arrows point to neovascularization. The red arrows in CFA show calcification in the necrotic core in the vK-stained slide.

### UNICORN outperforms the transformer baseline and aligns with human expert attention

UNICORN is evaluated in a 5-fold cross-validation scheme, splitting the dataset on a patient level into 60/20/20 train/validation/test sets for each fold (see “Methods” for details). All segments belonging to a patient are added to the same fold to avoid information leakage and as different sections of a patient may belong to different classes, the class distribution may not be consistent across the data splits. We compare our network with simpler approaches that accept all four stains as a single concatenated input (Table 2). Unlike our model, the baseline models do not embed each stain individually. To account for variability in the data splits, we perform four repetitions of 5-fold cross-validation for each of the models. The performance of each model is thus an aggregate over 20 data points. Our model achieves an average F1-score of 0.66 ± 0.04 (mean ± standard deviation) and an accuracy of 0.68 ± 0.05 compared to an F1-score of 0.62 ± 0.05 and an accuracy of 0.64 ± 0.06 for the second-best model, which is a simple transformer. All baselines are trained with the same data splits, and input data obtained from the same feature extractor. The F1-score is a macro average over all classes.

**Table 2:** UNICORN outperforms state-of-the-art MIL models on multi-stain coronary artery classification

	F1-score	Accuracy
AttentionMIL^15^	0.41 ± 0.05	0.51 ± 0.05
Perceiver^32^	0.53 ± 0.06	0.57 ± 0.07
Transformer^11^	0.62 ± 0.05	0.64 ± 0.06
UNICORN	**0.66** ± **0.04**	**0.68** ± **0.05**

UNICORN is significantly better than AttentionMIL (F1-score *p* = 1.9e-6, Wilcoxon-signed-rank tests, Accuracy *p* = 1.9e-6), Perceiver (F1-score *p* = 1.9e-6, Accuracy *p* = 1.9e-6), and transformer (F1-score *p* = 0.0003, Accuracy *p* = 0.004). For all baseline models, patches from the different stains are concatenated into a single input. Mean and standard deviation are calculated over four repetitions of a 5-fold cross-validation. Bold values indicate the best results.

**Fig. 2:**
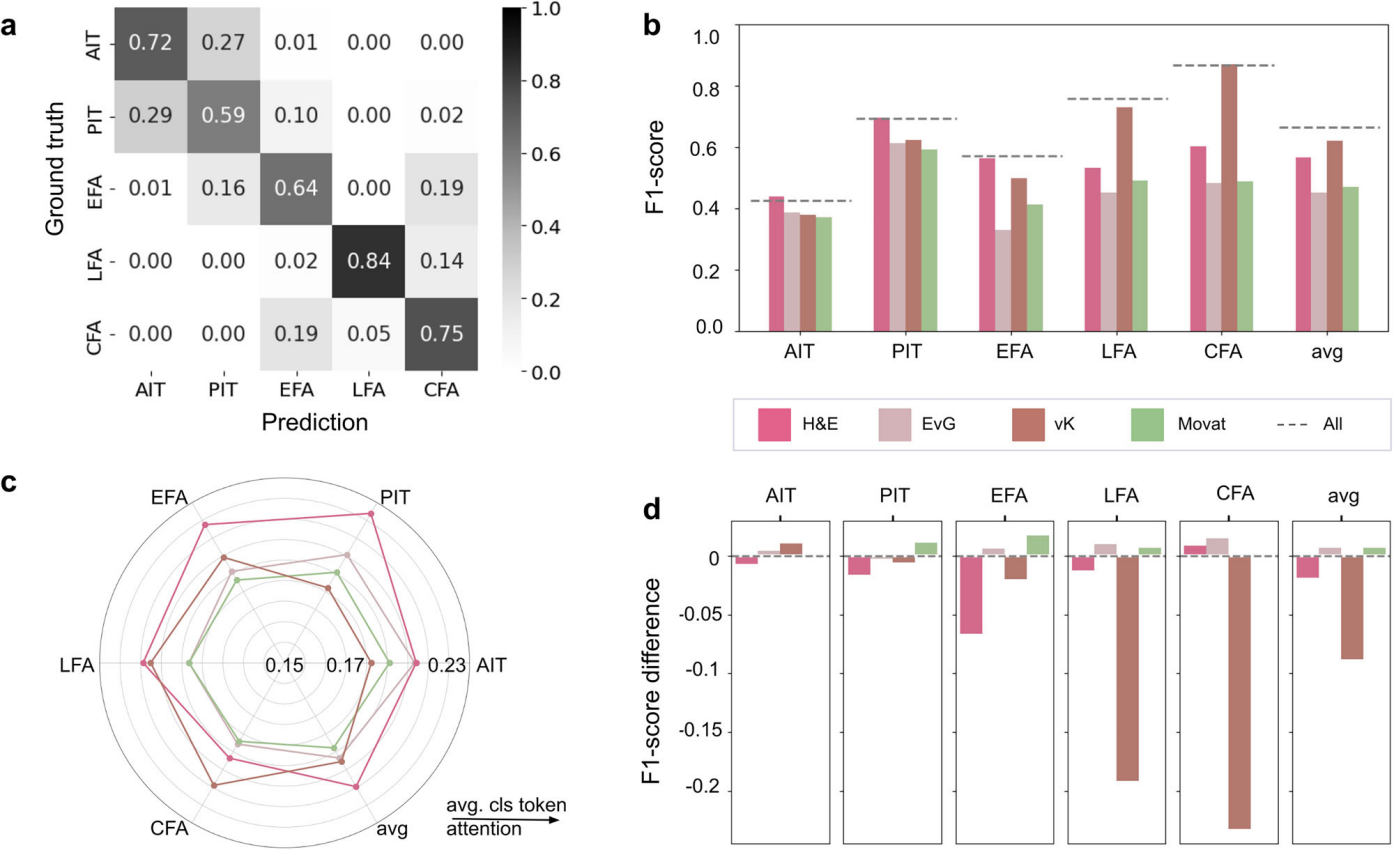
UNICORN integrates information from four stainings. **a** UNICORN discriminates multi-stain atherosclerosis WSIs into five classes with high accuracy. Accuracy is color-coded from white (0) to black (1). **b** Performance of UNICORN using just one staining as input, with other stains masked. The highest F1-score is achieved when using all four stainings (gray dotted line), indicating that UNICORN successfully aggregates information of the different stainings. **c** The attention mechanism of the UNICORN aggregation expert model gives higher attention values for stainings exerting a strong influence on performance for a given class. The mean attention value of the CLS token to the four stain tokens corresponding to a given staining is shown. **d** F1-score difference when utilizing three out of four stainings vs. all stainings (gray dotted zero line), bar colors indicate which staining was excluded. Results reported are from one of the four repetitions of the 5-fold cross-validation experiment as described in Results. Supplementary Fig. 2 provides an overview of performance across all repetitions and all stain combinations.

UNICORN outperforms the transformer model in per-class F1-score, precision, and recall for the classes PIT, LFA, and CFA (Table 3). For EFA, both models achieve similar recall, but UNICORN achieves better F1-score and precision. The transformer is marginally better across all metrics for AIT. Most misclassifications are observed between adjacent disease stages, which are inherently difficult to classify and distinguish even for highly trained experts (Fig. 2a). Our model demonstrates strong performance with single-staining inputs, but achieves optimal results when aggregating the information from all stainings (Fig. 2b and Supplementary Fig. 2). It also outperforms an ensemble of fours models trained with single stains (see ablation study results in Supplementary Table 1). When using only one staining as input for UNICORN, the vK staining performs best in classifying CFA and LFA, and second-best on EFA. H&E staining works best for AIT, PIT and EFA. Movat and EvG perform roughly on par across classes (Fig. 2b).

**Table 3:** Per-class metrics of UNICORN outperform the simple transformer baseline for every class but AIT

Metric	Model	AIT	PIT	EFA	LFA	CFA
F1-score	Transformer	**0.45**	0.67	0.53	0.69	0.83
	UNICORN	0.43	**0.69**	**0.57**	**0.76**	**0.87**
Precision	Transformer	**0.32**	0.89	0.45	0.7	0.83
	UNICORN	0.30	**0.84**	**0.52**	**0.77**	**0.89**
Recall	Transformer	**0.78**	0.54	**0.64**	0.68	0.83
	UNICORN	0.72	**0.59**	**0.64**	**0.75**	**0.84**

Metrics are calculated based on the predictions on samples from the test set. Since each sample (i.e., a unique combination of patient and artery segment) can be tested only once in a cross-validation setup, mean and standard deviation values for these metrics do not exist. Bold values indicate the best results within each combination of class and metric.

The attention of the CLS token to the stain-specific token in the expert model aggregation (Fig. 2c) demonstrates that the model focuses on the most relevant staining for its predictions. In Fig. 2d, one staining is left out for inference to visualize the difference in accuracy compared to inference with all four stainings. The results align well with previous experiments: Excluding von Kossa silver stain reduces the classification performance in advanced phenotypes (LFA, CFA), while H&E is the most important staining for the remaining classes. The results presented in Fig. 2b–d illustrate the relevance of the different stainings and the underlying biological processes. For instance, the vK staining, which imparts a black chromatic hue to calcifications, is of particular importance for the identification and differentiation of the calcified fibroatheroma (CFA) and lipid-rich late fibrous atheromas (LFA). As illustrated in Fig. 2b and c, H&E staining is fundamental for evaluating cell densities and general tissue structure. This is, the most crucial aspect for differentiating AIT and PIT, which represent different stages of atherogenesis, with variations in cell density, inflammatory infiltration, and extracellular matrix organization. Removing EvG or Movat has little effect and sometimes even improves performance in a single cross-validation experiment. However, consolidating the results over all four repetitions of the training shows that, on average, performance drops in comparison to using all four stains (Supplementary Fig. 2).

Overall, leaving out one stain (see four dark blue bars in Supplementary Fig. 2) causes the F1-score to drop by 2.3% on average. Leaving out a combination of two stains (six blue bars in Supplementary Fig. 2) drops performance by 6.3%, while leaving out three stains (colored bars) leads to a drop of 13.5%.

We compare the classification performance of UNICORN with that of a third biomedical expert (MvS), who is different from the two experts who labeled the ground truth. The test dataset contains 15 tissue samples chosen randomly to represent the class distribution of the original data. On this subset, UNICORN achieves an accuracy of 0.80 and a macro average F1-score of 0.80. In contrast, the expert achieved an accuracy of 0.53 and an F1-score of 0.48. Considering the limited size of the test dataset and inter-expert variability, we think that this comparison should be interpreted with care, but it clearly demonstrates the potential of UNICORN as an assistive diagnosis tool for expert pathologists.

To quantify the alignment of the human expert’s attention with that of UNICORN, we employ a simple evaluation scheme: For each stain of each sample in the test set, we show the expert (MvS) the WSI marked with five randomly ordered patches, containing two high attention, and three low attention patches (see examples in Supplementary Fig. 3). The expert’s task is to choose two patches out of the five that are considered to be most relevant to classify the image. The test set consists of 59 WSIs in total (15 samples with 4 stains each; one stain missing for one sample). In 41 (69.5%) WSIs, the expert chose those two patches to be most relevant, that got a high attention in the UNICORN classification model, indicating a good level of alignment. The expert and UNICORN disagreed completely (i.e., none of the two high attention patches were chosen by the expert as being relevant) in 5 (8.5%) WSIs. In the remaining 13 (22%) WSIs, the expert chose one of the two high attention patches. Movat pentachrome and EvG slides have the highest level of alignment, while von Kossa shows the least alignment among the stains (see detailed distribution of alignment across stains in Supplementary Fig. 4).

### UNICORN highlights explainable features

**Fig. 3:**
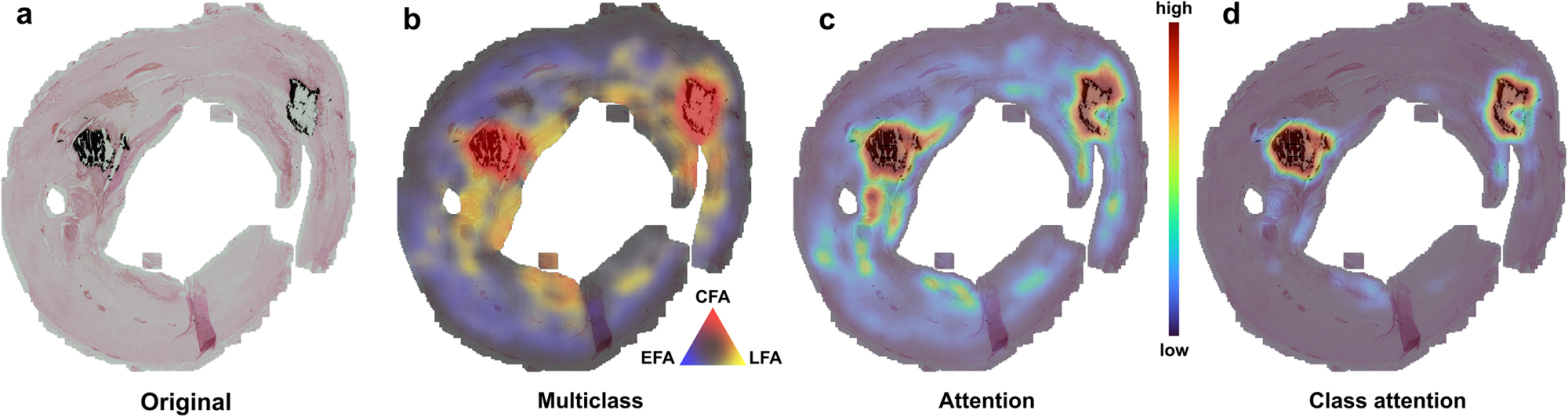
High-resolution heatmaps highlight relevant regions for classification decisions. **a** Original von Kossa silver stain image showing black calcification regions. **b** Coloring indicates the presence of structures associated with the most severe disease classes (EFA = blue, LFA = yellow, CFA = red). **c** Attention scores (lower score = blue, high score/high importance = red) show high attention regions. **d** Multiplication of attention score by the probability of the class that is predicted by UNICORN illustrates which regions are considered as important .

Three different visualization methods are provided to improve explainability based on the attention mechanism, resulting from an attention rollout^24^ over both stages of the transformer (Fig. 3). Figure 3b illustrates a tissue phenotype visualization, where tissue regions are highlighted based on the model’s interpretation of the class most indicative of each area (see “Methods”). Due to practical reasons, in this case, only the three most progressed disease tissue types are considered. Figure 3c highlights areas of attention computed by attention rollout independently per staining. Figure 3d combines the information of the first two methods into “class attention,” which highlights the tissue phenotypes that the model identifies with high attention and associates with its predicted class. In conclusion, three meaningful visualization maps are given: (i) “Where does the model attend to?” (ii) “Where does the model find phenotypes of the respective classes?”, and (iii) “Where does it attend to the predicted class?” which in our experiments highlights the areas of interest best and which are used in the following figures. These visualizations improve the interpretability of the model’s decisions, allowing researchers and clinicians to map the model’s predictions to specific tissue regions, and increase confidence in the model by transparently highlighting its decision-making process.

**Fig. 4:**
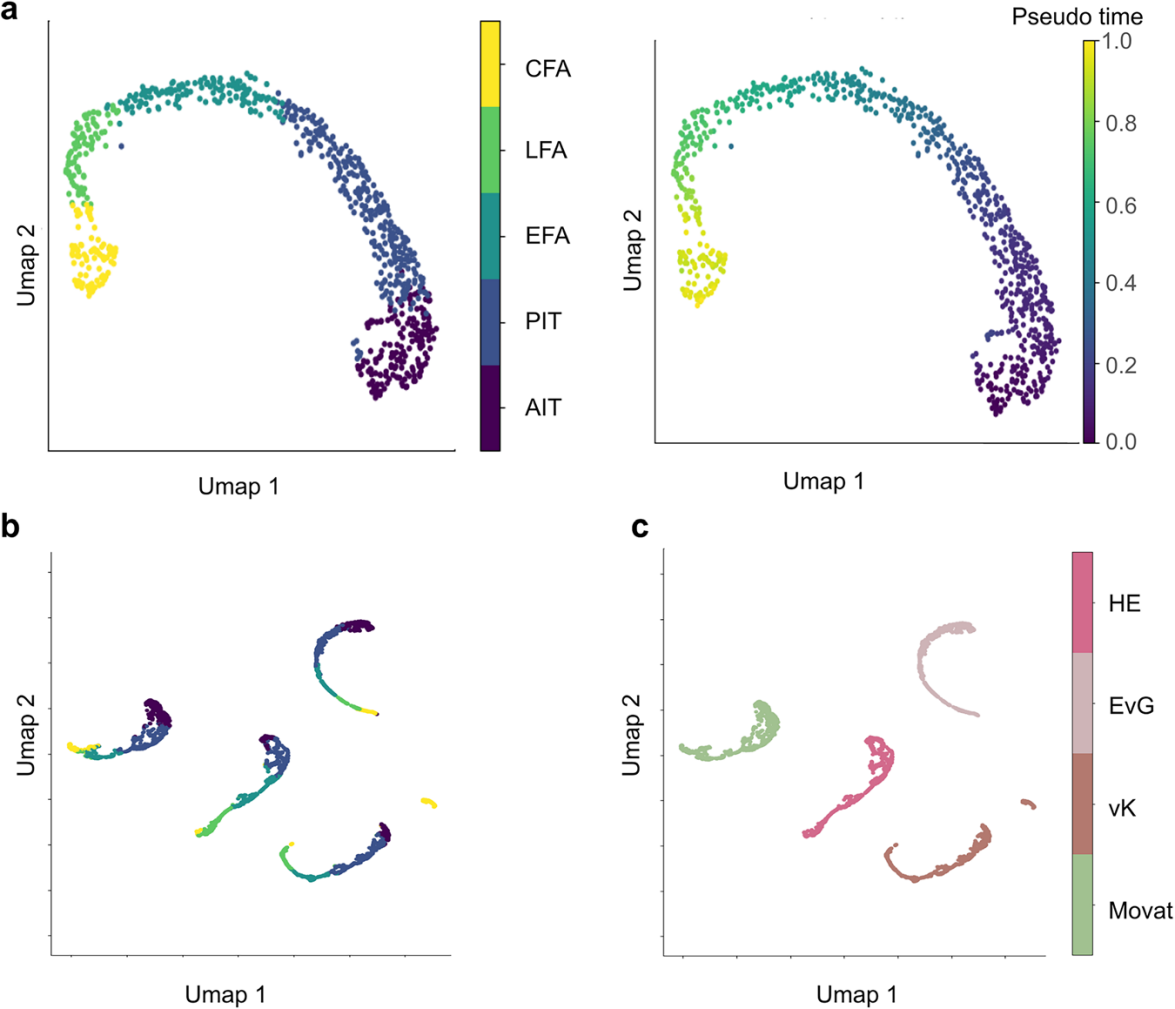
UMAP reveals disease progression modeling capabilities. **a** UMAP (left) and pseudotime trajectory analysis (right) of features of the final layer. **b** Lesions are similarly ordered when only one staining is used as input. **c** The model is able to distinguish which staining is processed.

Figure 4a illustrates that UNICORN is capable of accurately modeling the natural progression of atherosclerosis, as evidenced by a UMAP of an additional internal dataset comprising 768 segments from 114 individuals. The pseudotime trajectory analysis^25–27^ uses scanpy 1.11.3 (pseudocode in Supplementary materials). The capacity of UNICORN to capture this progression implies that the model is not only performing classification but is also learning a representation that aligns with the underlying biological process of the disease. This suggests that the model’s decision-making is grounded in clinically relevant features, thereby enhancing its utility in both diagnostic and research settings. Furthermore, the progression model exhibits consistency when considering individual stainings, as shown in Fig. 4b. The model can still provide meaningful insights even when only partial data is accessible, which is common in real-world clinical environments. This ability to generalize to a single staining suggests that the features learned by the model are rooted in the tissue's biological characteristics rather than artifacts of the specific staining technique. As illustrated in Fig. 4c, the model’s capacity to differentiate between various stainings is supported by empirical evidence. This ability to distinguish the particular staining processing is a crucial step towards effectively handling missing data, as it inherently recognizes what information is absent.

**Fig. 5:**
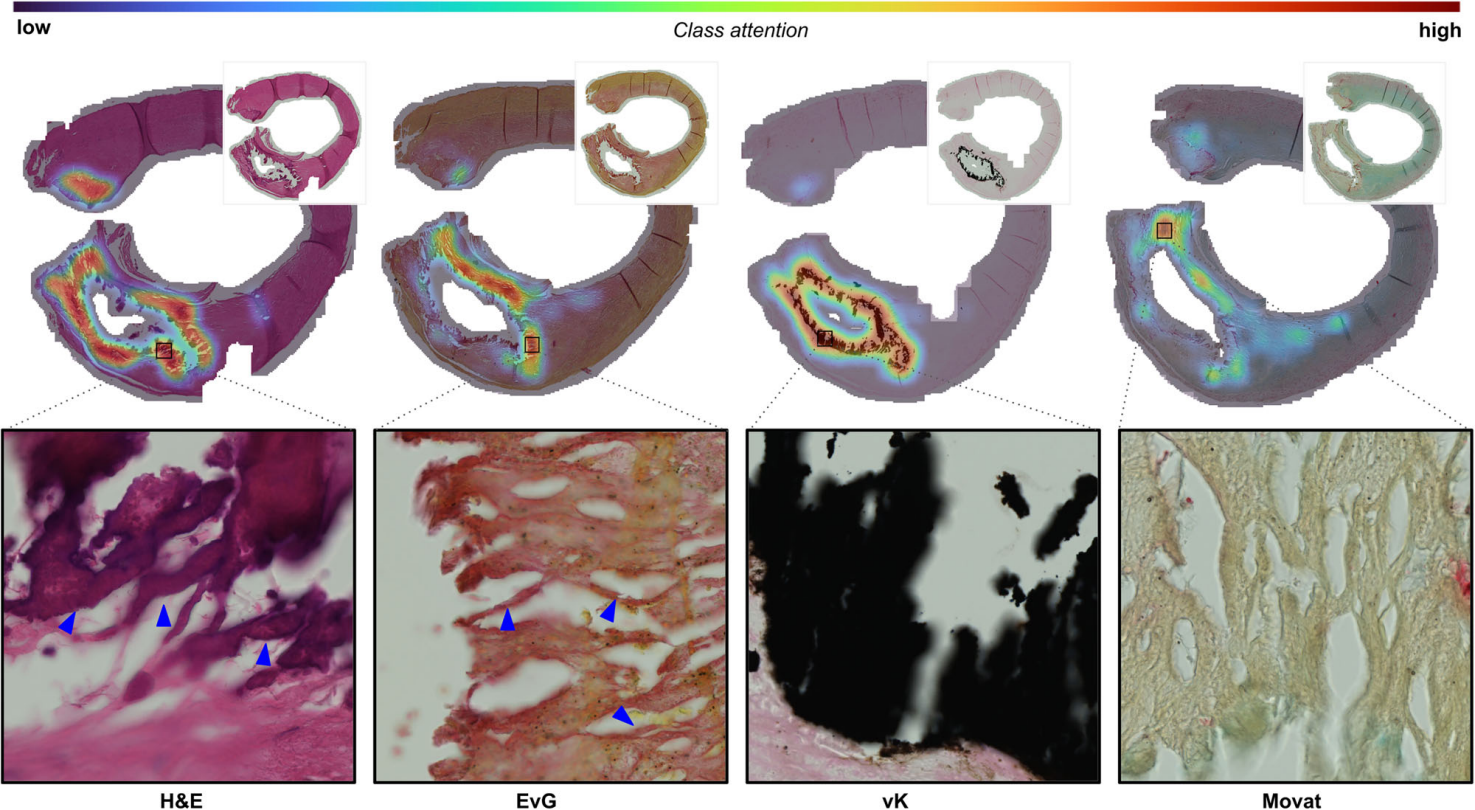
UNICORN highlights stain-specific classification of relevant regions. Highest class attention regions are shown in red across the four different stainings (H&E, EvG, vK, Movat) for a calcified fibroatheroma (CFA) case. The enlarged regions show the highest class attention regions. For H&E and EvG, the most relevant structures are highlighted with blue arrows. The highlighted patches in vK reveal the presence of calcified deposits within the intima. Other stains reveal a lipid-rich necrotic core, with extracellular lipid deposits and cellular debris. A fibrous cap composed of smooth muscle cells, collagen, and other extracellular matrix components covers the necrotic core.

Figure 5 shows, as an example for the CFA class, class attention maps and corresponding high class attention patches according to Fig. 3d, revealing regions identified by UNICORN as critical for classification. Additional examples for AIT, PIT, EFA and LFA cases are shown in Supplementary Fig. 1a–d. The high-class attention patches in the CFA case demonstrate the most relevant regions for the phenotype learned by the model and closely match the expert opinion. The sections show a lipid-rich necrotic core consisting of extracellular lipid deposits and cellular debris across the four different stains. The necrotic core is covered by a fibrous cap composed of smooth muscle cells, collagen and other extracellular matrix components.

In the H&E-stained sections, the algorithm focuses on regions characterized by dense fibrotic areas and calcified necrotic cores (blue arrows), which are typical of advanced fibroatheromas. These areas, highlighted in purple, correlate with expert annotations marking regions of calcification and fibrosis. In the EvG stain, the algorithm detects regions rich in extracellular matrix components, particularly elastic and collagen fibers, shown as red wavy structures (blue arrows). These structures, particularly in the shoulder region, outer necrotic core and intima, are critical in distinguishing CFA from earlier lesion stages, as CFA indicates less extracellular matrix due to remodeling in the vessel wall. Von Kossa (vK) staining accentuates calcifications, and the model places particular attention on black-stained regions, indicating areas of significant calcium deposition within the intima. This focus assists in differentiating CFA, in which calcification is a defining feature. Finally, the Movat pentachrome stain highlights the lipid-rich necrotic core in yellow-green, which the algorithm correctly identifies as a key pathological feature of CFA. By focusing on these areas, UNICORN demonstrates its ability to distinguish CFA from less advanced fibroatheromas, and to accurately identify relevant histologic features across multiple staining techniques.

## Discussion

We present UNICORN, a transformer-based model designed to analyze multi-stain histopathological data. In comparison to a simple transformer, UNICORN achieves better results and more importantly, UNICORN provides additional insights into the importance of the different stains for different stages of atherosclerosis. As precision medicine and personalized healthcare advance, the model’s ability to synthesize information from diverse sources makes it a valuable tool for research and clinical practice. UNICORN might augment pathologist workflows by providing explainable preliminary assessments and highlighting critical areas of interest in tissue samples, thereby expediting the diagnostic process. Its ability to perform inference on only a subset of possible stains can reduce the need for additional staining, optimizing resource use and cost.

The UNICORN framework holds promise for application beyond coronary artery disease. UNICORN achieves an F1-Score of 0.69 and an AUC of 0.70 on the task to predict the results of neoadjuvant chemotherapy on an HER2-positive breast cancer dataset^28^ without additional image preprocessing or changes to the framework, which is encouraging (see Supplementary materials). Thus, the UNICORN architecture is adaptable to other disease phenotypes and could be augmented by integrating high-throughput functional or longitudinal data. This integration would offer deeper insights into disease mechanisms and support more precise phenotyping. Future research could also investigate replacing initial expert models with alternative networks, to further enhance model performance. Additionally, incorporating clinical and high-throughput data into the model could provide a more comprehensive understanding of disease-specific pathology and functional biology, and improve classification accuracy.

Despite its promising capabilities, UNICORN has several limitations. The model's performance depends on the quality and diversity of the staining protocols used. In the absence of the most effective stain for a given class, the performance is capped by the performance of the next most informative stain. In some cases, where specific stains (such as Movat or Elastica van Gieson) are less informative when other stains are present, the model’s effectiveness may be reduced (Fig. 2d). Notably, the current feature extractor CTransPath^29^, trained on H&E-stained images only, may not fully leverage information from other stains, potentially limiting the model’s overall performance. Future work should explore fine-tuning strategies and the integration of alternative foundation models to address this issue. A similar trend is observed in our preliminary results on the HER2-positive dataset^28^ presented in the Supplementary materials (Supplementary Table 2). Furthermore, generalizability to other phenotypes and the integration of multi-omics data remain to be validated.

Another important factor is the bias introduced by the demographics of atherosclerosis patients represented in our dataset. Apart from the fact that most individuals are older, there is also a substantial imbalance in the male-to-female ratio. This can bias the model against the range of age, sex, race, and other demographic factors not represented in the dataset. Dependence on the facility creating the digitized slides can also affect the features extracted. Strategies to mitigate bias introduced by hardware used and other variations introduced during the procurement pipeline need to be implemented before the model can be deployed. Computational resource requirements must also be considered for clinical deployment. UNICORN has 12.1M trainable parameters in comparison to 2.5M for simple transformer, 7.1M for Perceiver, and 231k for AttentionMIL. Finally, this work is still in the proof-of-concept stage. Practical implementation in a clinical setting will require significant investments in digitization of existing workflows, data curation, software design and user training.

In conclusion, the UNICORN model represents a significant advancement in the integration and analysis of multi-stain histopathological data. Its ability to handle missing stains and provide comprehensive insights across diverse staining techniques positions it as a valuable tool for enhancing diagnostic accuracy and efficiency. While the model shows promising results in atherosclerosis classification, further optimization and validation are needed to fully realize its potential across various disease phenotypes. The integration of UNICORN into research and clinical workflows has the potential to significantly improve diagnostic consistency and support explainable personalized medicine.

## Methods

### Histological data compilation, selection, and staining

The Munich cardIovaScular StudIes biObaNk (MISSION) was launched in 2019 and comprises seven cardiovascular-relevant tissue samples from each individual, such as coronary and carotid artery tissue samples, as well as myocardium. It also includes blood and plasma, liver, skeletal muscle and various adipose tissues from more than 1000 deceased individuals, collected in formalin for FFPE sections (formalin fixed paraffin embedded) and fresh frozen at −80 °C. Tissue sections were deparaffinized in xylene substitute and rehydrated through a graded alcohol series. After curating the dataset to exclude 5 samples with stains of poor quality, a total of 1045 tissue samples from 177 individuals were analyzed in this study, covering the following coronary arteries: each proximal and distal RCA (right coronary artery), the LAD (left anterior descending artery) and the LCX (left circumflex coronary artery) as well as the LM (left main) (Fig. 1a). Each tissue section was subjected to a series of histopathological analyses, including hematoxylin and eosin (H&E) staining, Elastica van Gieson (EvG) staining, von Kossa (vK) silver staining, and Movat Pentachrome (Movat) staining. These techniques were used to assess histopathological features of atherosclerotic lesions.

The histological classification of the atherosclerotic lesions was performed in accordance with the American Heart Association (AHA) classification and the adapted classification system proposed by Virmani et al.^3^. The lesions were categorized into the following five stages (Fig. 1b). Advanced intima thickening (AIT) is defined by diffuse intimal thickening composed of smooth muscle cells and extracellular matrix in the absence of significant lipid accumulation or inflammatory infiltration. Pathological intimal thickening (PIT) is primarily characterized by the accumulation of extracellular lipid and inflammatory cells within the intima, with a necrotic core absent. For the vulnerable stages, early, late, and calcified fibroatheroma (EFA, LFA, and CFA) are distinguished. Early fibroatheroma (EFA) is distinguished by a thin fibrous cap and a developing lipid core, whereas the late fibroatheroma (LFA) is more advanced, exhibiting a larger necrotic core and a thicker, though still potentially unstable, fibrous cap. Additionally, LFA displays greater inflammatory involvement. Calcified fibroatheroma (CFA) represents the late stage of fibroatheroma development, with microscopical and macroscopical calcification present within the necrotic core. The fibrous cap may be thickened, and the lesion exhibits reduced cellularity due to extensive calcification.

The institutional review board and ethics committee of the Technical University of Munich, Germany approved our protocol of the MISSION Biobank for postmortem tissue (2018-325-S-KK-22.08.2018). Postmortem samples were collected only after release by the public prosecutor and with consent from the next of kin. Individuals known to have expressed opposition to organ donation or research participation during their lifetime were excluded. The study was performed in accordance with the provisions of the Declaration of Helsinki and the International Conference on Harmonization guidelines for good clinical practice.

### Image patching and feature extraction

A publicly available pipeline from Wagner et al.^11^ was used to extract patches and the corresponding features from the WSIs. As the feature extractor, we use CTransPath^29^, which has been trained on H&E TCGA and PAIP datasets. We apply CTransPath to each staining without additional finetuning or stain normalization to investigate its performance without expending additional computational resources on stain normalization and without introducing stain normalization biases. The resulting 256 × 256 px patches are extracted without overlap for training. Blurred regions and uncolored background are excluded using a Canny edge detection algorithm^30^ with a threshold of one edge to be detected per patch. A 5× resolution for atherosclerosis prediction was used, as it has been empirically shown to perform best.

### Model training

UNICORN is trained for 30 epochs using an AdamW^31^ optimizer. The learning rate and the weight decay are set to 2.0e-5. Gradients are accumulated across 16 batches with a batch size of 1 before an update step is made. To increase robustness and force the model to be able to handle missing data, it is trained with domain dropout of 0.7, where domains are randomly masked and not used as input. During training, for each input, one of the input domains is randomly chosen that will not be masked; all other stainings or additional domains have a chance of 0.7 to be masked. The stains that have not been masked are input to the respective expert model, and the outputs of these expert models are passed to the aggregator model. The pseudocode is as follows:

#### Algorithm 1:

Mask input stains with dropout probability p


def generate_dropout_list(x, p):


 n ← length of x

 values ← list of n False values

# Select one random index to ensure at least one True

 randint ← random integer between 0 and n−1

 values[randint] ← True

# Ensure selected element is valid (not None or empty)

if x[randint] is None OR number of elements in x[randint] is 0:

# Retry with new random selection

return generate_dropout_list(x, p)

# Iterate through remaining indices

for i from 0 to n−1:

if values[i] is False:

# Set to True with probability p

if random number between 0 and 1 < (1−p):

   values[i] ← True

return values

#### Algorithm 2:

UNICORN forward pass with masked input


def forward(x):


 x ← list of feature tensors from multiple sources (e.g., stains)

 x_agg ← empty list of aggregated inputs

if test_mode is False:

  dropout_list ← generate_dropout_list(x, stain_dropout)

else

  dropout_list ← [True, True, …, True] - length as x

 for i ← 0 to length(x) − 1 do

 x_i ← x[i]

if x_i is not None or empty and dropout_list[i] is True:

   x_i_transformed ← expert_model[i](x_i)

Append x_i_transformed to x_agg

# x_agg only contains non-masked, non-zero transformed stains

 x_input ← concatenate(cls_tokens, x_agg)

 x_transformed ← aggregator_transformer(x_input)

 x_norm ← layer_norm(x_transformed)

 x_out ← Linear(x_norm)

return x_out, x_norm

Since the model is transformer-based, it is inherently capable of processing multiple inputs of varying lengths. As the loss function, weighted cross-entropy is used to account for class imbalance.

### Model attention visualization

To get high-resolution attention maps, slides are patched with 95% overlap between patches, after which features are extracted. This results in 400 distinct feature bags, each representing the full slide. Each of these bags is independently forwarded through the network. The attention that is generated by attention rollout^24^ on the expert models for each of the patches for each bag is all saved. Additionally, for each bag, only a single patch is forwarded, and the model predicts a class based on one patch only, which results in the class scores. Using these class scores, one can visualize the Multiclass visualization (Fig. 3b) by multiplying the color corresponding to a certain class by the class probability for a patch. Multiplying the attention scores by the multiclass score of the predicted class then gives the class attention score. In the end, the scores across overlapping patches are averaged for the detailed maps. All scores are normalized between 0 and 1.

## Supplementary information

Supplementary Materials

## Data Availability

In accordance with data privacy laws, human data from the MISSION Biobank cannot be made publicly available, but can be requested by qualified researchers by contacting M.v.S. at the German Heart Centre. Requests may be subject to the submission and approval of a research proposal and a data transfer agreement.

## Code availability

The code for the UNICORN multi-stain model is available at https://github.com/ValentinKoch/UNICORN. This repository includes the environment specification, scripts for training and inference, and a README. The scripts for processing the WSIs and feature extraction are available at https://github.com/peng-lab/HistoBistro/tree/feature_extraction.
