# Supplementary material for: UNICORN: a deep learning model for integrating multi-stain data in histopathology
Source: NPJ Digit Med. 2026 Jun 18;9:480. doi: 10.1038/s41746-026-02829-6 (PMC13287598; doi:10.1038/s41746-026-02829-6)
Supplement: Supplementary file 1 — Supplementary Materials [file 41746_2026_2829_MOESM1_ESM.pdf]

# Supplementary materials

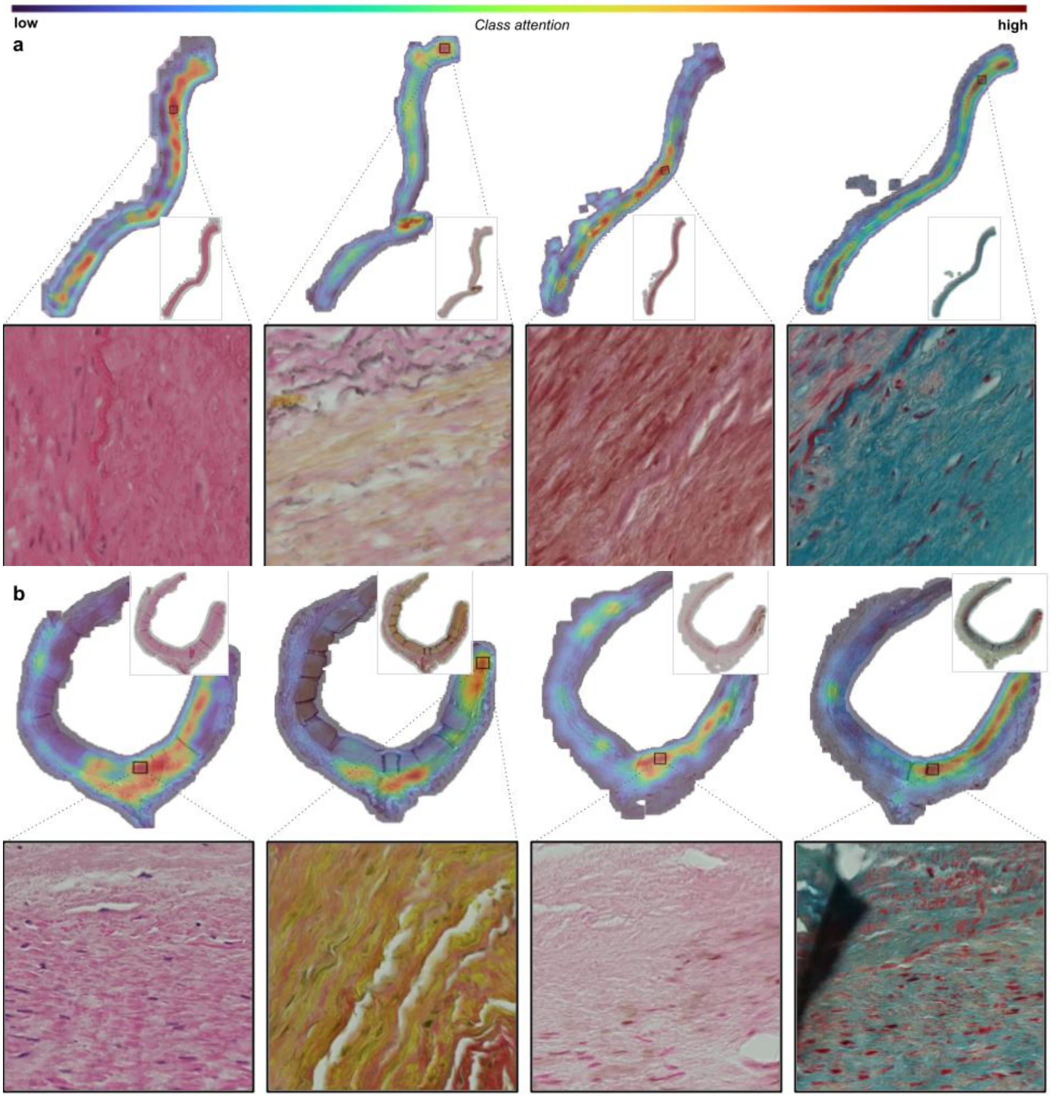

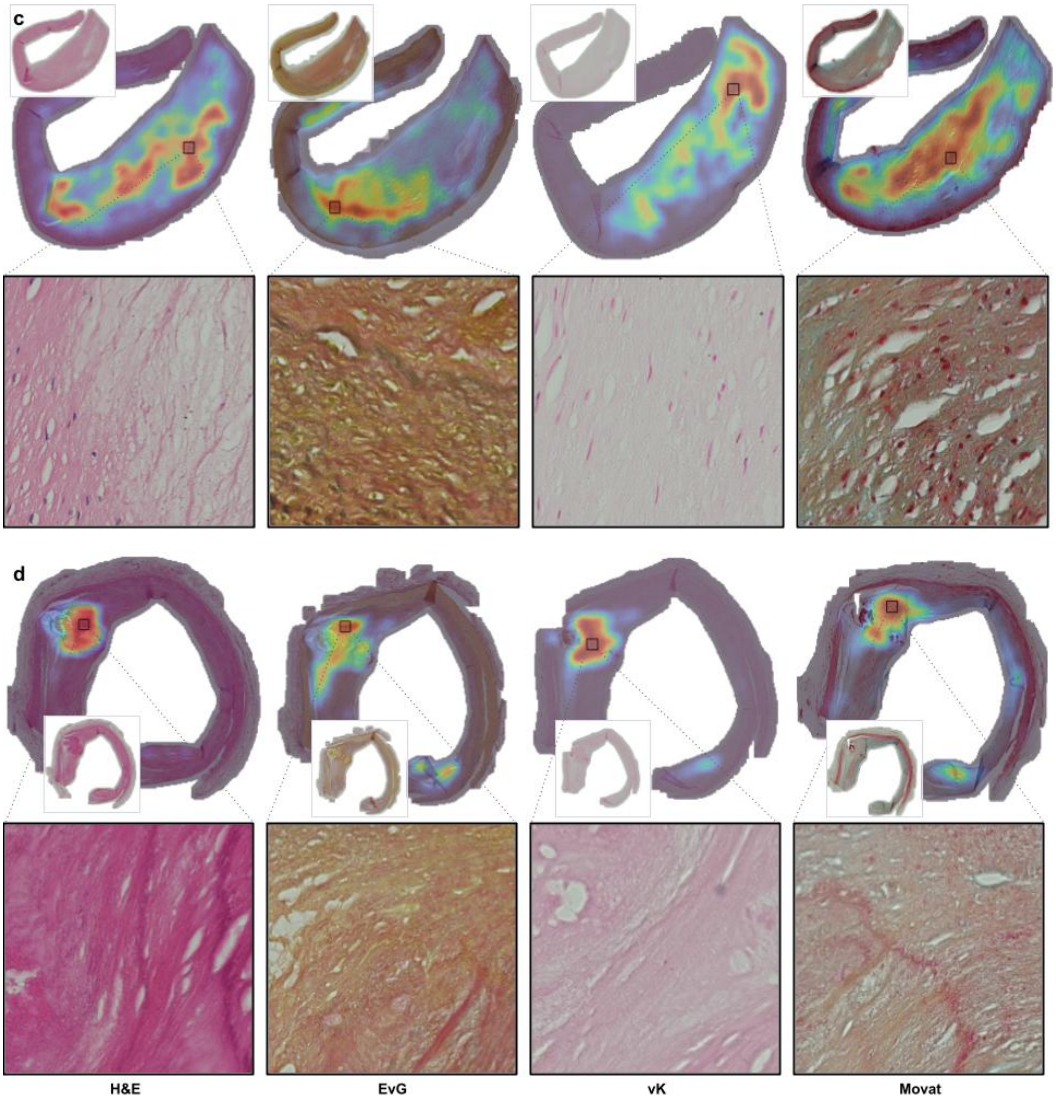


**Supplementary Figure 1**
a: UNICORN effectively identifies and classifies adaptive intima thickening (AIT) using four distinct stainings: Hematoxylin and Eosin (H&E), Elastica van Gieson (EvG), von Kossa, and Movat Pentachrome (Movat). The prioritized patches with the highest score illustrate thickening of the intima with intact cell structures, highlighting the accumulation of smooth muscle cells and extracellular matrix within the arterial wall's intimal layer.
b: Pathologic intima thickening (PIT). The highlighted patches reveal increased numbers of smooth muscle cells within the intima, enhanced deposition of extracellular matrix components such as collagen and proteoglycans, the presence of extracellular lipid pools or droplets within the intimal layer without the formation of a necrotic core, and absent inflammatory cell infiltration.
c: Early fibroatheroma (EFA). The highlighted patches reveal a well-defined lipid-rich necrotic core within the intima, consisting of extracellular lipid deposits and cellular debris. A fibrous cap, composed of smooth muscle cells, collagen, and other extracellular matrix components, covers the necrotic core. There is a presence of macrophages and foam cells at the edges of the necrotic core, along with initial signs of inflammation indicated by some infiltration of inflammatory cells, though not as prominent as in advanced lesions.
d: Late fibroatheroma (LFA). The highlighted patches reveal a prominent, well-defined lipid-rich necrotic core consisting of extracellular lipid deposits, cholesterol crystals, and cellular debris. This core is covered by a thick fibrous cap composed of layers of smooth muscle cells, collagen, and other extracellular matrix components. The presence of macrophages, foam cells, and other inflammatory cells is noted particularly at the edges of the necrotic core and within the fibrous cap.

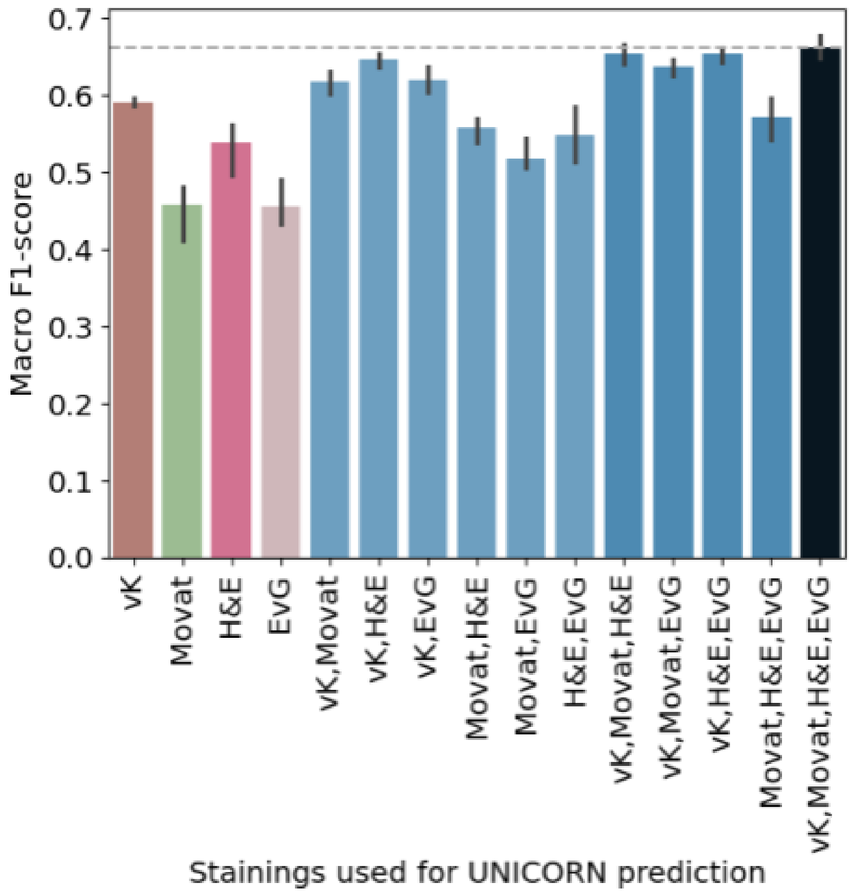


**Supplementary Figure 2: Prediction using all four stains achieves the highest F1-score among all stain combinations.**

Error bars represent the minimum and maximum values achieved across inference runs with all possible stain combinations on models obtained from four 5-fold cross-validation training runs with different seeds.

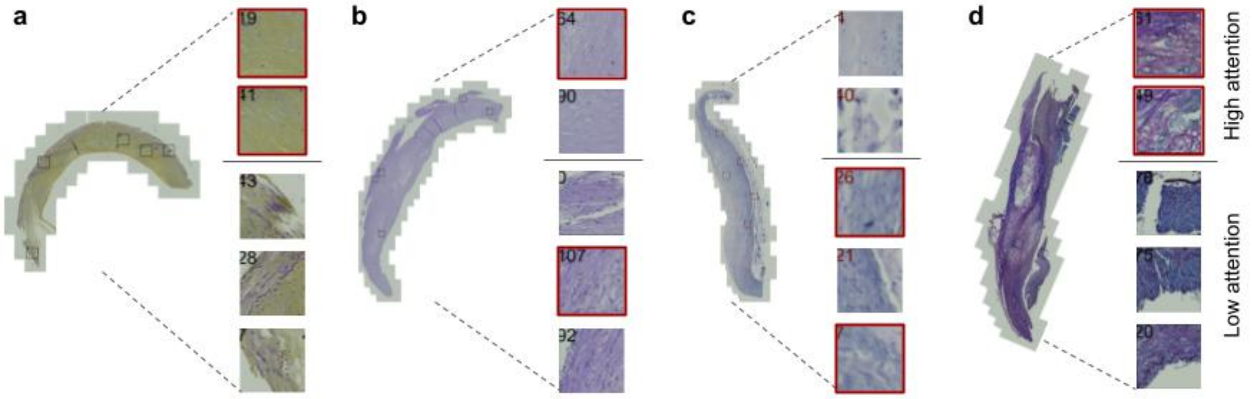


**Supplementary Figure 3: UNICORN high attention patches are considered to be relevant for classification by the human expert.**
We randomly select 15 samples, each consisting of one WSI for each stain. From each WSI, we select two patches with the highest attention, and three with the lowest attention. Four exemplary WSIs are shown here. The human expert sees the full WSI and the five patches (without attention information) marked on the WSI. Zoomed in images of the patches marked for the expert are displayed: two high attention (above the separator) followed by three low attention patches (below the separator). The patches with red borders are the ones selected by the expert as being relevant to classify the WSI.
a: Movat pentachrome stain where both high attention patches were selected by the expert.
b: H&E stain where one of the two high attention patches matched with the expert's assessment.
c: Von Kossa stain where none of UNICORN's two high attention patches are marked as relevant by the expert.
d: EvG stain where the expert selected both high attention patches.

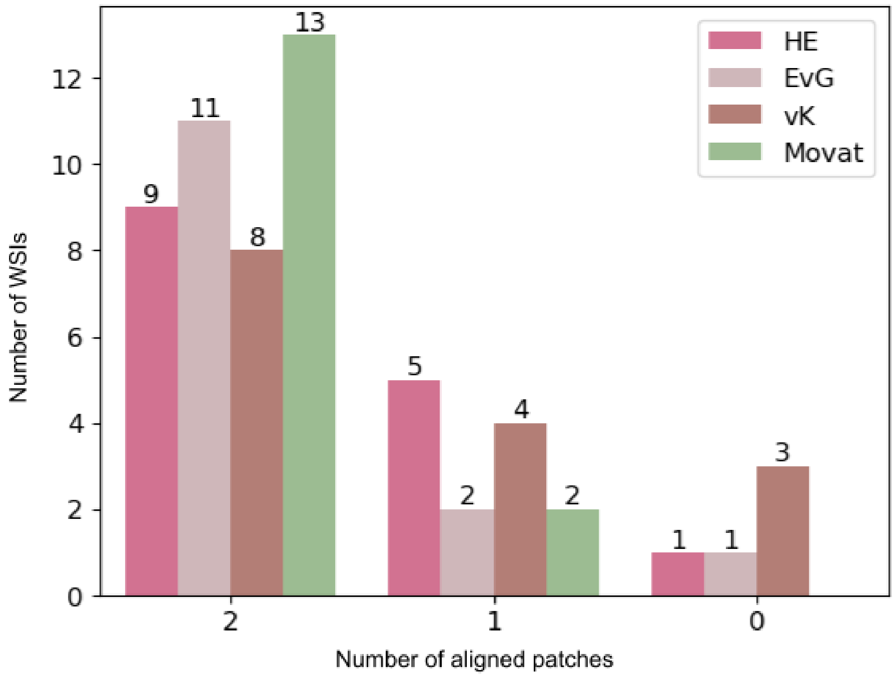


**Supplementary Figure 4: UNICORN achieves highest alignment with expert on patch attention for Movat pentachrome stain.**
Stain-wise distribution of the alignment between UNICORN high attention patches and patches that are considered relevant by an expert for the test set of 59 WSIs. Expert is shown two high attention, and three low attention patches per WSI (see Supplementary Figure 3). The expert chooses two patches out of the five that are most relevant for classification.

## Trajectory analysis for disease progression

We perform a trajectory analysis to show that the features used by UNICORN for classification inherently model the progression of coronary atherosclerosis from adaptive intima thickening (AIT) to calcified fibroatheroma (CFA). We use the Python package scanpy 1.11.3 to perform this analysis. Scanpy is a toolkit built originally for single-cell gene expression analysis including trajectory inference. Specifically, we use the *scanpy.tl.dpt* method to perform diffusion pseudotime[30,31] to infer the progression from UMAP data.

We perform the analysis by running inference on 768 artery segments from 114 patients separate from the patients used for training and validation. The features from each segment from the last layer of the model before the fully connected layer are used to perform the analysis. We compute a UMAP for these features, which in turn is used to compute a nearest neighbor map, followed by a diffusion map[32]. In this diffusion map space, given a root cell (in our case the label AIT as it is the first stage of disease progression), the diffusion pseudotime method orders the trajectory based on geodesic distances.The pseudocode for the full process is as follows:

```
Algorithm S1: Trajectory analysis
________________________________________________________________
def trajectory_analysis(features, labels):
    adata ← load features and labels (n_obs × n_vars, labels)

    # Compute nearest neighbors (nn) distance matrix and graph
    nearest_neighbors(adata, method='umap')

    # Embed nn graph with UMAP
    umap(adata)

    # Calculate diffusion pseudotime
    # Set segments with label AIT as the initial state
    adata.uns['iroot'] ← adata[label == 0]
    dpt ← diffusion_pseudotime(adata)
    plot(dpt)
```

## UNICORN ablation study

To assess the contribution of components of the model architecture, we perform an ablation study. We first compare the performance of UNICORN on a single stain to the performance of a “single-stain” model, trained with only one stain, for each of the four stains (see model architecture in Supplementary Figure 5). We then evaluate the performance of UNICORN on a multi-stain image against a hard voting and soft voting ensemble of the four single-stain models, i.e. we consider the majority vote of the models without any weighting. While single-stain models outperform UNICORN on single stain inference, UNICORN outperforms the ensemble of single-stain models when inference is performed on the multi-stain images from our test data.

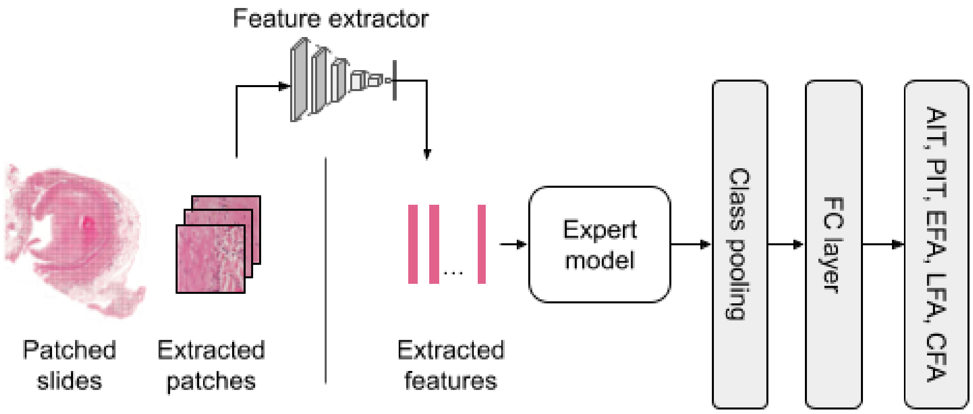


**Supplementary Figure 5: A "single-stain" model is trained with only one stain for the whole dataset.** We train in total four "single-stain" models, one for each of the staining techniques (H&E, von Kossa, Movat pentachrome, and EvG). The figure shows the "single-stain" model for H&E.

**Supplementary Table 1. UNICORN outperforms the ensemble of single-stain models.** Macro averaged F1-score and accuracy are calculated based on the predictions made on samples (a single-stained image in the upper part, a multi-stain image for the ensemble comparison) from the test set. All models were trained on the same data splits.

| Model | F1-score | Accuracy |
|---|---|---|
| Single-stain H&E | 0.54 | 0.57 |
| UNICORN H&E | 0.57 | 0.60 |
| Single-stain EvG | 0.53 | 0.57 |
| UNICORN EvG | 0.46 | 0.49 |
| Single-stain vK | 0.63 | 0.63 |
| UNICORN vK | 0.62 | 0.62 |
| Single-stain Movat | 0.52 | 0.54 |
| UNICORN Movat | 0.47 | 0.49 |
| Single-stain ensemble (hard voting) | 0.62 | 0.66 |
| Single-stain ensemble (soft voting) | 0.62 | 0.64 |
| UNICORN | **0.66** | **0.67** |

## Results on a multi-stain breast cancer dataset

To test the model's generalizability to non-coronary tissue, we train UNICORN to predict the outcome of neoadjuvant chemotherapy on an external multi-stain, breast cancer dataset. Huang et al. present a special feature extraction method called IMPRESS, which performs better than hand crafted features by pathologists in predicting the outcome of neoadjuvant chemotherapy (NAC) on HER2-positive (HER2+) breast cancer patients[25]. Their method involves the registration of immuno-histochemistry (IHC) whole slide images to H&E slides, followed by segmentation of H&E slides into tumor, stroma, and lymphocyte aggregated regions, while the IHC slides are segmented into cell markers CD8, CD163, and PD-L1. A combination of these tissue-level features results in 36 tabular IMPRESS

features, which along with six clinical features are used to predict outcome in the form of pathologic complete response (pCR, 0 or 1) using logistic regression. The main contribution of Huang et al. is the sophisticated feature extraction, whereas our method relies on pre-trained feature extractors used out of the box.

To test UNICORN's applicability, we use CTransPath[26] to extract features from the H&E and IHC slides, which are then passed through our pipeline consisting of two expert models to generate the stain tokens, followed by an expert model aggregator, class pooling and MLP, as described in Fig. 1. The clinical features are concatenated in the input to the MLP. Given the small dataset size (62 samples), we perform leave-one-out cross validation, similar to the original work. UNICORN achieves an F1-Score of 0.69 and an AUC of 0.704 (see Supplementary Table 2). Although UNICORN does not outperform IMPRESS, we argue that the results are promising, given that our model does not require pre-processing such as image registration and segmentation. Notably, the IHC domain is very different to the H&E domain on which the feature extractor is originally trained, possibly limiting the extraction of stain specific features.

**Supplementary Table 2. UNICORN shows promising performance on an external, non-coronary dataset.** IMPRESS is a multistep method that involves registration, tissue annotation and segmentation, feature extraction and incorporation of clinical data. The authors report mean and standard deviation over 20 repetitions of a leave-one-out cross validation[25]. We report macro averaged values from a single experiment with leave-one-out cross validation.

| | AUC | F1-Score | Precision | Recall |
|---|---|---|---|---|
| IMPRESS | **0.898 ± 0.004** | **0.869 ± 0.008** | **0.872 ± 0.012** | **0.866 ± 0.008** |
| UNICORN | 0.704 | 0.690 | 0.694 | 0.704 |

We test the performance of UNICORN with missing stains by masking a stain at random according to the logic explained in Algorithm 1. This is possible due to the inherent capability of UNICORN to train and infer despite missing stains. We train the model for masking probabilities between 0.4 (lower chance) to 0.7 (higher chance) in steps of 0.1 of one of the two stains getting masked. None of the clinical features are masked. As expected, the performance drops increasingly across all metrics (Supplementary Figure 6). For the highest masking probability of 0.7, the AUC drops by 15% whereas the F1-Score drops by 18%.

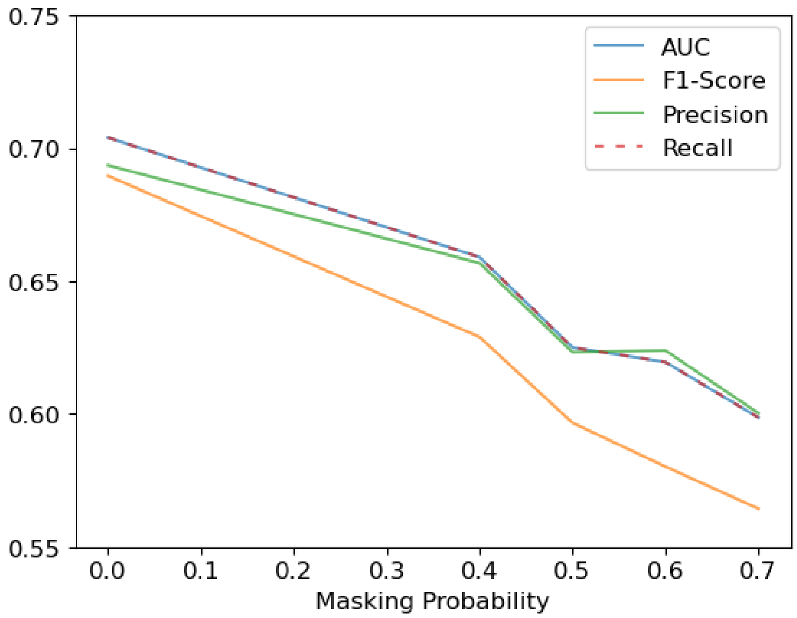


**Supplementary Figure 6: At 0.7 masking probability, AUC drops by 15% and F1-Score drops by 18%.** We train UNICORN on the HER2+ dataset with stain masking probabilities from 0.4 (low chance) to 0.7 (high chance). At least one stain is always passed as input while the other stain is masked according to the masking probability. Performance drops across all metrics with higher masking probability.
